# DnaK1‐mediated regulation of phototactic motility in *Synechococcus elongatus* UTEX 3055

**DOI:** 10.1002/mlf2.70109

**Published:** 2026-10-26

**Authors:** Shang‐Yu Li, Qing‐Xue Sun, Dan‐Qing Huang, Tao Xie, Yan Yu, Chenliu He, Cesar A. Valades‐Cruz, Xiaoli Zeng, Yiling Yang, Cheng‐Cai Zhang

**Affiliations:** ^1^ Institute of Hydrobiology Chinese Academy of Sciences Wuhan China; ^2^ University of Chinese Academy of Sciences Beijing China; ^3^ Hubei Hongshan Laboratory Wuhan China; ^4^ State Key Laboratory of Lake and Watershed Science for Water Security Chinese Academy of Sciences Nanjing China; ^5^ Present address: School of Medical Technology Xinxiang Medical University Xinxiang China

**Keywords:** bacterial motility, cyanobacteria, DnaK, molecular chaperone, phototaxis

## Abstract

Cyanobacterial phototaxis relies on type IV pili (T4P); yet, how light signals are transmitted to the T4P motor remains unresolved. Here, we identify the molecular chaperone DnaK1 as an important regulator of phototactic motility in *Synechococcus elongatus* UTEX 3055. DnaK1 interacts with the phototaxis regulator PixG_Se_ and the T4P extension ATPase PilB, and loss of *dnaK1* impairs phototactic motility. Mutations in *dnaJ1* impair phototaxis, and a partially segregated *grpE* mutant shows a similar but milder defect, suggesting that the DnaK1–DnaJ1–GrpE system may contribute to this process. Fluorescence imaging shows that DnaK1, PixG_Se_, and PilB have similar polar localization patterns. Based on these findings, we propose a working model in which DnaK1 may facilitate the assembly or stability of phototaxis‐associated T4P complexes, thereby providing a potential mechanistic link between the phototaxis signaling module and T4P motor activity. These findings establish a link between DnaK1 and the phototaxis machinery, highlighting a potential role for molecular chaperones in coupling light‐sensing pathways to T4P‐mediated motility.

## Impact statement

This study shows that the chaperone DnaK1 promotes phototactic movement in cyanobacteria. DnaK1 connects PixG_Se_, a key signaling protein in the phototaxis pathway, to PilB, the motor that powers movement. When DnaK1 is removed, cells move less efficiently toward light. We propose that DnaK1 mediates the association of PixG_Se_ and PilB at the cell pole to enable light‐directed movement. While direct evidence is still needed, our findings raise the possibility that chaperones serve as organizers for motility‐related protein complexes, pointing to a broader role for these proteins in regulating bacterial movement beyond their well‐known function in stress responses.

## INTRODUCTION

Heat shock proteins (HSPs) are conserved molecular chaperones in both eukaryotes and prokaryotes that protect cells from environmental stresses such as heat, high light, and salinity by facilitating protein folding, disaggregation, and degradation^1^. In bacteria, the Hsp70 homolog DnaK, along with its co‐chaperones DnaJ (Hsp40) and GrpE (nucleotide exchange factor), forms the KJE system, which maintains protein homeostasis through an ATPase‐driven cycle^2^. The nucleotide‐binding domain (NBD) of DnaK alternates between ATP‐ and ADP‐bound states, while the substrate‐binding domain (SBD) of DnaK undergoes conformational changes controlled by ATP hydrolysis. These transitions are further modulated by DnaJ (Hsp40) and GrpE^3^. Beyond stress protection, the KJE system also regulates the assembly and disassembly of protein complexes, highlighting broader roles for chaperones in cellular regulation^4^.

Cyanobacteria, oxygenic photoautotrophs central to global carbon and nitrogen cycles, encode multiple DnaK homologs with distinct functions (Table 1). In *Synechococcus* sp. PCC 7942 (hereafter PCC 7942), three DnaK paralogs, DnaK1, DnaK2, and DnaK3, show distinct expression patterns and roles. DnaK2 is strongly induced by heat, high light, or salt stress, functioning as the canonical system for protein folding; DnaK3 localizes to the thylakoid membrane, potentially aiding protein translation^7^, ^12^, ^13^, ^14^. DnaK1, though nonessential for growth, displays a low salt‐dependent ATPase activity and is less characterized^22^. Similarly, in *Synechocystis* sp. PCC 6803, DnaK1, DnaK2, and DnaK3 have distinct roles (Table 1). DnaK2 is critical for stress adaptation, while the role of DnaK1 remains elusive^5^, ^9^, ^10^, ^23^. Unlike most bacteria, which encode a single DnaK, the presence of multiple DnaK homologs in cyanobacteria suggests functional diversification; yet, their specific contributions are poorly understood.

**Table 1:** DnaK, DnaJ, and GrpE homologs in *Syenchocystis* sp. PCC 6803, *S. elongatus* PCC 7942, and *S. elongatus* UTEX 3055.

	PCC 6803		PCC 7942		UTEX 3055
Gene name	Gene ID	Description	Gene ID	Description	Gene ID
*dnaK1*	sll0058	Nonessential; involved in regulation of motility^5^, ^6^	Synpcc7942_2073	Nonessential; no heat shock response^7^, ^8^	**RS08885**
*dnaK2*	sll0170	Essential; typical heat shock response; expression under circadian control^5^, ^9^, ^10^, ^11^	Synpcc7942_2468	Essential; typical heat shock response; regulating RNA degradation through interaction with RNaseE^7^, ^8^, ^12^	RS06710
*dnaK3*	sll1932	Essential; attaching to membrane surface^5^	synpcc7942_2580	Essential; no heat shock response; assisting protein translation on the thylakoid membrane surface^7^, ^13^, ^14^, ^15^	RS06110
*dnaJ1*	sll0897	Nonessential; canonical heat shock protein^16^	Synpcc7942_2074	Essential^17^	**RS08880**
*dnaJ2*	slr0093	Nonessential^16^	Synpcc7942_1789	Essential; heat shock protein; cytosol^17^	RS10325
*dnaJ3*	sll1933	Essential^16^	Synpcc7942_2579	Essential^18^	RS06115
*dnaJ4*	sll1666	Nonessential^16^	Synpcc7942_0266	Nonessential^17^	RS04600
*dnaJ‐*like	sll0909	Nonessential^16^	Synpcc7942_0666		RS01955
*dnaJ‐*like	sll1384	Nonessential, phototaxis, RbcL assembly^16^, ^19^, ^20^	Synpcc7942_0812		RS01475
*dnaJ‐*like	sll1011	Nonessential^16^	Synpcc7942_2306		RS07530
*dnaJ‐*like			Synpcc7942_1467		RS11150
*dnaJ‐*like			Synpcc7942_2389		RS07105
*dnaJ‐*like			Synpcc7942_2536		RS06370
*grpE*	sll0057	Canonical heat shock protein; interacting with all three DnaK proteins^10^, ^21^	Synpcc7942_2072		**RS08890**

Gene IDs analyzed in this study are highlighted in bold.

Many cyanobacteria show phototaxis, a type IV pili (T4P)‐mediated movement toward or away from light. T4P are dynamic surface appendages that drive twitching or gliding motility and are also required for DNA uptake and biofilm formation^6^, ^24^, ^25^. The dynamic assembly of T4P mainly depends on the motor ATPase PilB, which promotes the secretion of the pilin protein PilA, whereas PilT catalyzes pilus retraction through PilA recycling. In *Synechocystis* sp. PCC 6803, positive phototaxis is regulated by a two‐component system that links photoreceptors to T4P activity, with the response regulator PixG_Se_ acting as a key mediator through direct interaction with PilB^26^, ^27^. Additional components at the pilus base are also crucial for assembly and regulation. For example, Hfq and EbsA form a tripartite complex with PilB, which controls multiple pilus‐related processes, including twitching motility, DNA competence, and flocculation^28^, ^29^, ^30^. These findings reveal the pilus base as a regulatory hub integrating signaling and structural components.

Recent studies have implicated DnaK chaperones in pilus‐dependent processes. In the filamentous cyanobacterium *Nostoc punctiforme*, DnaK1 interacts with PilB and Hfq, and is required for secretion of hormogonium polysaccharide (HPS), but does not directly regulate T4P activity or phototaxis^31^. Thus, while the precise function of DnaK1 appears to differ between species, with polysaccharide secretion in *Nostoc* versus a potential role in phototaxis in unicellular cyanobacteria, the observation that DnaK1 can physically associate with the T4P motor is at least conserved.

In the rod‐shaped, unicellular cyanobacterium *Synechococcus elongatus* UTEX 3055, phototaxis is mediated by a chemotaxis‐like system that converges on the response regulator PixG_Se_ ^32^. However, the downstream interaction partners of PixG_Se_ have not been systematically identified, and it remains unknown whether a DnaK family chaperone participates in this process. Given that DnaK1 can interact with PilB in other cyanobacteria^26^, ^31^, we hypothesize that DnaK1 might function at the pilus base to integrate environmental signals—such as light—into motility control. This conceptual framework guided our research design, focusing on whether DnaK1 interacts with known phototaxis regulators or T4P motors and whether such interactions are functionally required for phototactic motility.

Here, we investigate the function of DnaK1 in *S. elongatus* UTEX 3055 (hereafter UTEX 3055), a close relative of PCC 7942 that retains both phototaxis and biofilm formation.^33^ We demonstrate that DnaK1 physically interacts with PixG_Se_ and the T4P motor ATPase PilB. Loss of *dnaK1* impairs phototactic motility, suggesting a role for this chaperone in light‐responsive movement. Based on these observations, we propose a working model in which DnaK1 may be a part of a complex critical for phototactic motility. Our findings point toward a chaperone‐mediated mechanism that could regulate T4P activity and provide new insights into the ecology and adaptive behavior of cyanobacteria.

## RESULTS

### PixG_Se_ interacts with DnaK1

Phototaxis in cyanobacteria is mediated by a signal transduction system that couples light sensing to the T4P motility apparatus, enabling directed cell movement. PixG_Se_ is a key regulator of this process, linking the phototaxis system to the motility machinery, likely through interaction with PilB^26^, ^27^, ^32^. However, the mechanistic detail of this regulation remains unclear. To identify novel interaction partners of PixG_Se_, we performed co‐immunoprecipitation (Co‐IP) assays using a strain in which Flag‐tagged versions of PixG_Se_ were expressed in a *pixG* mutant background. Both the N‐ and the C‐terminal tagged fusion proteins (3× Flag‐PixG_Se_ and PixG_Se_‐3× Flag, respectively) restored phototaxis in the *pixG* mutant, though the N‐terminal tagged variant showed more robust complementation than the C‐terminal tagged one, and was thus selected for further analysis (Figure 1A).

**Figure 1:**
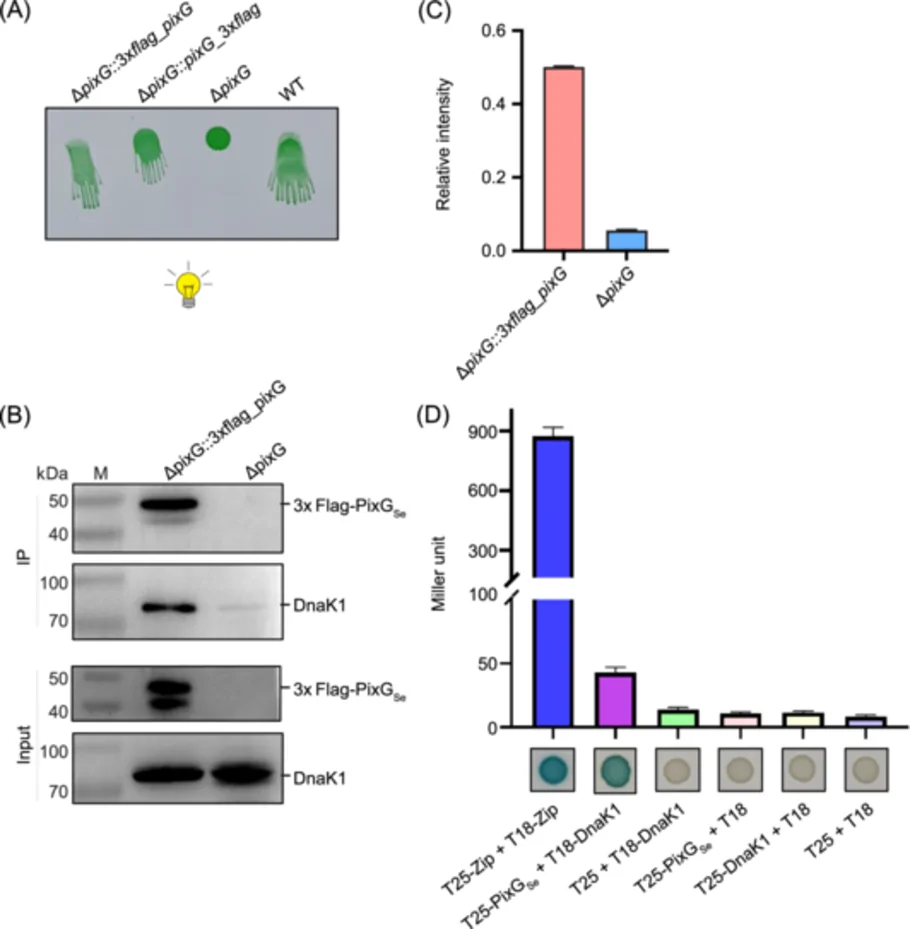
Identification of DnaK1 as an interaction partner of PixG_Se_. (A) Phototaxis behavior of wild‐type (WT), Δ*pixG* mutant, and complemented strains (Δ*pixG*::3×*flag_pixG*) under leaky expression conditions (without IPTG). Fresh cell cultures (OD_750_ = 7.0) were spotted (2 µl) onto BG‐11 plates solidified with 0.4% (w/v) agarose and supplemented with 10 mM sodium thiosulfate. Plates were incubated for 5–7 days at 30°C under lateral illumination from a white LED lamp (30–80 μmol photons m^−2 ^s^−1^). The direction of light is indicated by a light bulb. (B) Co‐immunoprecipitation (Co‐IP) of DnaK1 with PixG_Se_ from the Δ*pixG*::3×*flag_pixG* strain using anti‐PixG_Se_ and anti‐DnaK1 antibodies. (C) Quantification of copurified DnaK1 in Δ*pixG*::3×*flag_pixG* and Δ*pixG* strains from (B). Band intensities were normalized to the input and expressed as fold enrichment relative to Δ*pixG*. (D) β‐galactosidase activity of PixG_Se_ and DnaK1 interactions measured using the bacterial adenylate cyclase two‐hybrid (BACTH) assay, expressed in Miller units (*n* = 3 biological replicates, two‐sample *t*‐test, *p* < 0.05). The positive control strain contains T25‐Zip and T18‐Zip, and the negative control strain contains T25 with T18 alone.

Cells expressing 3× Flag‐PixG_Se_ were cultured, and cell extracts were prepared and used for Co‐IP assays using anti‐Flag Magnetic Beads for affinity‐based protein purification. The Δ*pixG* strain was used as a control. Mass spectrometry (MS) analysis of the eluted proteins identified some proteins with high enrichment scores, including the known PixG_Se_ interactors PixL_Se_ and PixI_Se_‐2^32^, validating our approach. Strikingly, the most enriched hit RS08885 was a DnaK‐family protein, showing 182‐fold enrichment over the control (Table 2). Like PCC 7942, UTEX 3055 encodes three DnaK paralogs: DnaK1, DnaK2, and DnaK3. Sequence alignment with PCC 7942 homologs identified this enriched protein as DnaK1 (Figure S1). Immunoblotting with a DnaK1‐specific antibody confirmed its robust co‐purification with 3× Flag‐PixG_Se_, with minimal background in the control strain lacking tagged PixG_Se_ (Figure 1B,C).

**Table 2:** Proteins identified with co‐immunoprecipitation, followed by mass spectrometry (MS) analysis.

Gene ID	Enrichment fold	Annotation
RS08885	182.6	Molecular chaperone DnaK1
RS08825	57.5	AsrA Family ATPase
RS00020	36.2	Phosphomethylpyrimidine synthase
RS09210	24.0	AMC family ATPase
RS06465	20.2	ATP‐binding protein
RS01215	17.5	Chemotaxis protein CheW (PixI2)
RS01220	15.6	CheA‐like protein PixL
RS11900	13.7	NAD(P) transhydrogenase subunit alpha
RS07385	12.8	Glucose‐6‐phosphate dehydrogenase
RS12920	9.8	Molybdenum cofactor protein MoaC/MoaB
RS12290	9.8	2‐isopropylmalate synthase
RS01875	9.3	Glycosyltransferase family 9 protein

Given the high cellular abundance of DnaK and its potential for nonspecific binding, we further validated the PixG_Se_–DnaK1 interaction using a bacterial two‐hybrid system. A reproducible interaction was detected via β‐galactosidase activity assay, albeit weaker than the positive control (Figure 1D). This interaction was only observed when PixG_Se_ and DnaK1 were fused to the C‐termini of the T25 and T18 fragments, suggesting steric or conformational constraints. No interactions were detected between PixG_Se_ and other top candidates (except for the two known targets PixL_Se_ and PixI_Se_‐2) or the paralog DnaK2 (Figure S2A,B). Together, these results demonstrate a specific physical association between PixG_Se_ and DnaK1.

### Deletion of *dnaK1* impairs phototactic motility and enhances biofilm formation

Bioinformatic analysis revealed that *dnaK1* is organized in a potential operon structure flanked by genes encoding its canonical co‐chaperones DnaJ1 and GrpE, raising the possibility that these genes are co‐transcribed and functionally linked as part of the Hsp70 chaperone system (Figure 2A). To investigate their roles in phototaxis, we constructed deletion mutants of each gene in UTEX 3055. PCR‐based segregation analysis confirmed complete gene replacement for Δ*dnaK1* and Δ*dnaJ1* mutants, but repeated attempts to fully segregate the Δg*rpE* mutant were unsuccessful, as wild‐type copies persisted even after extended antibiotic selection (Figure 2A). This genetic evidence indicates that *grpE* is essential for viability, while *dnaK1* and *dnaJ1* are dispensable under standard conditions. The essentiality of *grpE* is explained by its unique, nonredundant role as the sole nucleotide exchange factor for all three DnaK paralogs in UTEX 3055. In contrast, the DnaK and DnaJ paralogs themselves have undergone functional diversification, some of which have evolved specialized, nonessential roles such as regulating motility, explaining their dispensability for growth under standard conditions (Table 1). Importantly, spot assays with cells on solid media showed no detectable growth defects for any of the mutants compared to the wild type (WT) (Figure S3).

**Figure 2:**
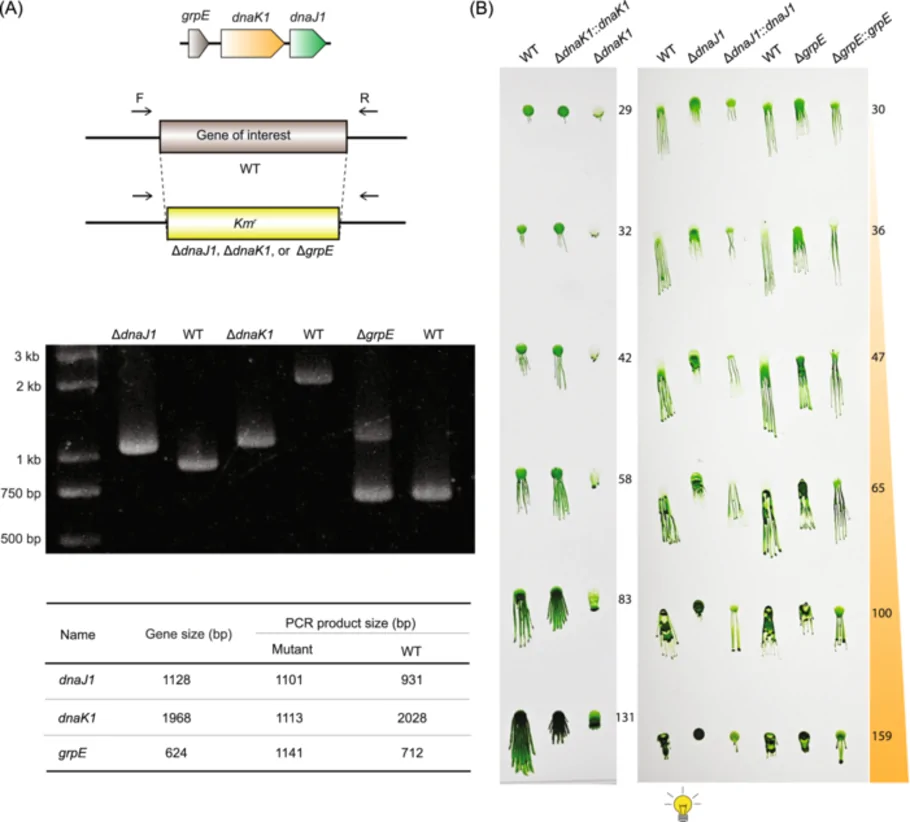
The DnaK1 chaperone system regulates phototaxis in UTEX 3055. (A) Genetic organization of the DnaK1–DnaJ1–GrpE (K1J1E) operon and verification of knockout mutants. Target genes were replaced with the *kanamycin*‐resistance (*km^r^*) cassette, and deletions were confirmed by PCR using flanking primers (F/R). Expected and observed amplicons sizes are summarized in the accompanying table. Note that the Δ*grpE* strain is not fully segregated, as a wild‐type copy of *grpE* remains detectable. (B) Phototaxis assays of WT, mutants (Δ*dnaK*, Δ*dnaJ1, and* Δ*grpE*), and complemented strains (expressed from the same locus under basal promoter activity without IPTG induction). Assays were performed as described in Figure 1A. A lateral white LED positioned at the bottom created a light gradient (orange triangle). Measured light intensities at defined positions along the plates ranged from 29 to 131 μmol photons m^−2^ s^−1^ (left plate) and from 30 to 159 μmol photons m^−2^ s^−1^ (right plate).

To test whether the DnaK1–DnaJ1–GrpE (K1J1E) system is involved in the specialized process of phototaxis, we analyzed the motility of the mutant strains under varying light intensities. The Δ*dnaK1* mutant showed severely reduced phototactic motility compared to WT across all light intensities tested (Figures 2B and S4). Quantitative analysis confirmed that Δ*dnaK1* showed significantly reduced migration compared to the complemented strain under all light intensities (Figure S4). The Δ*dnaJ1* mutant displayed similar defects, while the partially segregated *grpE* mutant showed only a mild, though statistically significant, impairment (Figure S4). In all cases, genetic complementation with the respective gene expressed from a P_*trc*_ promoter at a neutral genomic site (NS1) restored phototaxis to near wild‐type levels even without IPTG induction (Figures 2B and S4), confirming that the observed phenotypes were specifically caused by loss of these chaperone components, ruling out polar effects or secondary mutations.

Given prior reports that DnaK1 regulates extracellular polysaccharide production in *Nostoc* hormogonia^31^, and knowing that polysaccharides can contribute to both motility and biofilm formation in bacteria, we investigated whether *dnaK1* deletion affects biofilm development under standard growth conditions. The Δ*dnaK1* mutant showed an approximately fourfold increase in biofilm biomass compared to the WT, as quantified by crystal violet staining (Figure S5). The hyper‐biofilm phenotype suggests that DnaK1 may play a role in the transition between motile and sessile lifestyles, although direct evidence for this idea is not yet available. While the inverse relationship between reduced phototaxis and increased biofilm formation is consistent with a motile–sessile tradeoff, we note that our current data do not establish a direct mechanistic link between these two phenotypes. Possible explanations—including altered T4P activity, changes in extracellular matrix production, or direct effects on gene expression—remain to be tested.

### Pairwise interactions among DnaK1, PixG_Se_, and PilB

Genomic analysis revealed that in UTEX 3055, the *dnaJ1–dnaK1–grpE* chaperone gene cluster is located immediately upstream of a set of genes encoding key T4P components, including PilB, PilT1, and PilC (Figure 3A). This conserved genomic arrangement is observed in multiple cyanobacterial species, raising the possibility of an evolutionarily maintained linkage that may promote coordinated functions between the chaperone machinery and T4P systems (Figure S6). Functionally, targeted deletion of either *pilB* or *pilT1* completely abolished phototactic motility (Figure 3B), consistent with their essential roles in powering pilus extension and retraction, respectively, as reported^34^, ^35^, and thereby confirming that the genomic association corresponds to functionally critical components of the motility apparatus.

**Figure 3:**
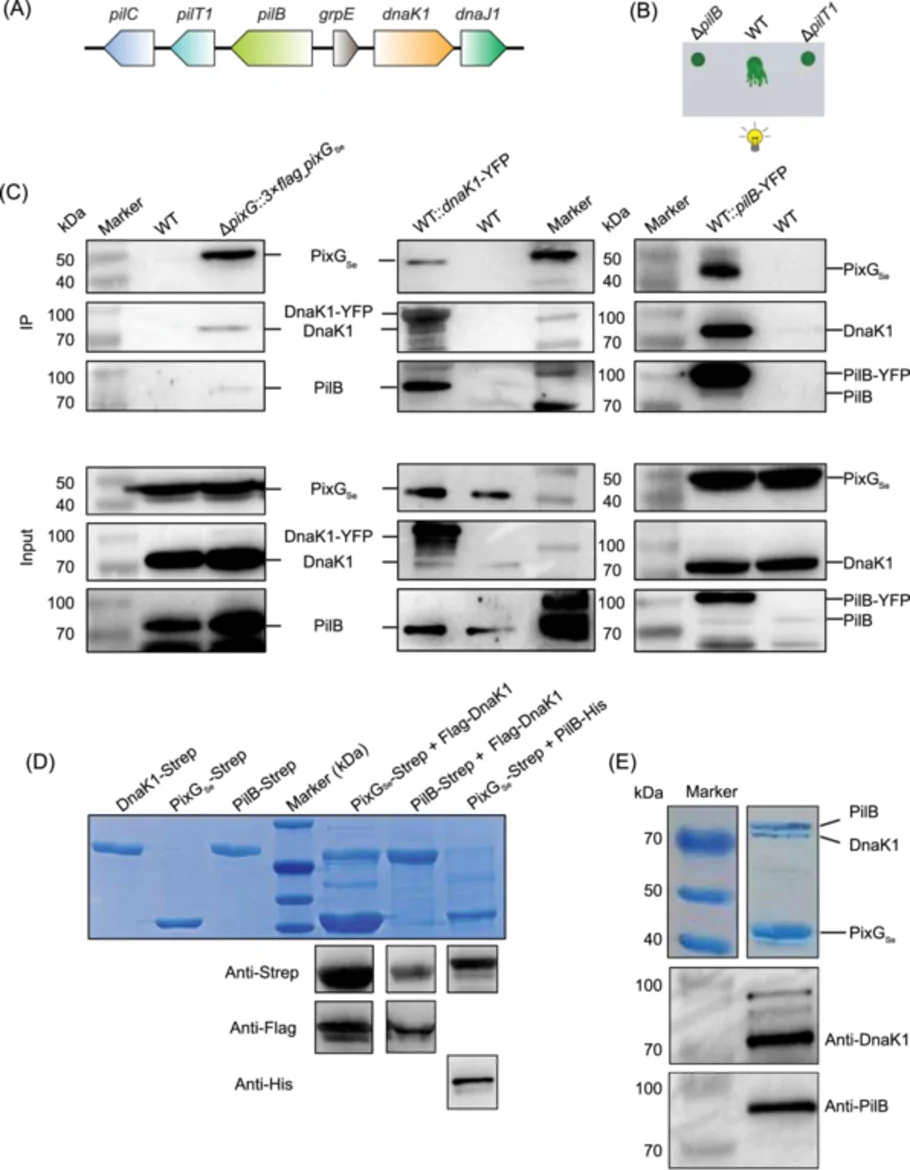
Pairwise interaction between DnaK1, PixG_Se_, and PilB. (A) Genomic organization of *pilC*, *pilT1*, *pilB*, *grpE*, *dnaK1*, and *dnaJ1* on the UTEX 3055 chromosome. (B) Phototaxis assays of WT, Δ*pilB*, *and* Δ*pilT1* strains. Cells were spotted on BG11 agarose plates (0.4%) and exposed to a lateral white LED lamp for 3–5 days. (C) Co‐IP of PixG_Se_, PilB, and DnaK1. Lysates from Δ*pixG::*3×*flag_pixG_Se_* (immunoprecipitated with anti‐Flag beads), WT::*pilB‐YFP,* and WT::*dnaK1‐YFP* (purified via GFP‐VHH agarose) were analyzed by Western blot using anti‐PixG_Se_, anti‐PilB, and anti‐DnaK1 antibodies, respectively. Input (supernatant) and eluted fractions are shown. (D) Pairwise interactions tested by co‐expressing Strep‐tagged PixG_Se_ with Flag‐tagged DnaK1, Strep‐tagged PilB with Flag‐tagged DnaK1, or Strep‐tagged PixG_Se_ with His‐tagged PilB in *Escherichia coli* BL21. Strep‐tagged proteins were affinity‐purified using Strep‐Tactin XT resin, and interactions were analyzed by SDS‐PAGE (Coomassie blue staining) and Western blot (gray panels). All three protein pairs (PixG_Se_–DnaK1, PilB–DnaK1, and PixG_Se_–PilB) co‐purified, demonstrating direct binding. (E) Validation of ternary complex formation. His‐PixG_Se_ was co‐expressed with untagged DnaK1 and PilB from a single plasmid in *E. coli* BL21. The His–PixG_Se_ complex was purified by Ni‐NTA affinity chromatography, and elution fractions were probed by Western blot using anti‐DnaK1 and anti‐PilB antibodies, confirming the presence of all three proteins in the complex.

To determine whether the chaperone system physically interacts with the phototaxis signaling protein PixG_Se_ and the T4P motor PilB, we carried out a series of Co‐IP experiments. We used specific antibodies against PixG_Se_, PilB, and DnaK1, expressing either Flag‐ or YFP‐tagged bait proteins in the corresponding deletion mutant or the WT background, with an untagged WT strain serving as a negative control. In Δ*pixG* lysates, Flag‐tagged PixG_Se_ efficiently co‐purified both endogenous DnaK1 and PilB (Figure 3C, left panel). Reciprocal Co‐IPs showed that YFP‐tagged DnaK1 pulled down PilB and PixG_Se_ (Figure 3C, middle panel), while YFP‐tagged PilB enriched both PixG_Se_ and DnaK1 in WT lysates (Figure 3C, right panel). Immunoblot analyses consistently detected two bands for YFP‐tagged PilB and DnaK1, one corresponding to the tagged version and one to the native untagged protein, demonstrating that these interactions occur between endogenous proteins rather than being artifacts of overexpression (Figure 3C).

To test whether these interactions are direct or require cyanobacterial‐specific factors, we reconstituted the protein associations in *Escherichia coli*. Pairwise co‐expression and pulldown assays revealed that 3× Flag‐DnaK1 co‐purified with both PixG_Se_‐Strep and PilB‐Strep, and that PilB‐His bound specifically to PixG_Se_‐Strep (Figure 3D). Furthermore, when all three proteins were co‐expressed, His‐PixG_Se_ simultaneously pulled down PilB and DnaK1 (Figure 3E), which is consistent with the possibility of a ternary complex. These findings were reproducible across multiple tag configurations and independent experiments, with appropriate controls confirming antibody specificity (Figure S7). Collectively, these data demonstrate physical interactions among DnaK1, PixG_Se_, and PilB. Based on these observations, we propose a model in which the DnaK1 chaperone is physically linked to the phototaxis regulator PixG_Se_ and the T4P motor PilB, potentially positioning it as a regulatory hub that integrates phototaxis signaling with motility machinery.

### Subcellular localization of the K1J1E system and T4P components

Domain analysis of the K1J1E chaperone proteins revealed conserved structural features consistent with their canonical functions. DnaJ1 contains an N‐terminal J‐domain required for interaction with DnaK^36^, as well as a zinc‐finger region defined by the conserved “CXXCXGXG” motif (Figure 4A). Phylogenetic analysis placed DnaJ1 closest to the class I DnaJ clade, most closely related to the *Synechocystis* protein Sll0897 (Figure S8), a canonical heat shock chaperone essential for growth under non‐optimal temperatures^16^. Notably, DnaJ1 is not an ortholog of the DnaJ protein influencing motility in *Synechocystis* (Sll1384) and *N. punctiforme* (Npun_F1160). DnaK1 comprises an N‐terminal nucleotide‐binding domain (NBD) and a C‐terminal substrate‐binding domain (SBD), connected by the conserved DVLLLD linker characteristic of the DnaK homologs. The SBD contains both a β‐sandwich and an α‐helical subdomain (Figure 4A), with the C‐terminal region predicted to mediate substrate recognition^37^. GrpE displays an unstructured N‐terminal segment, followed by α‐helical and β‐sheet regions (Figure 4A), consistent with its role as a nucleotide exchange factor that facilitates ADP release during the DnaK reaction cycle.

**Figure 4:**
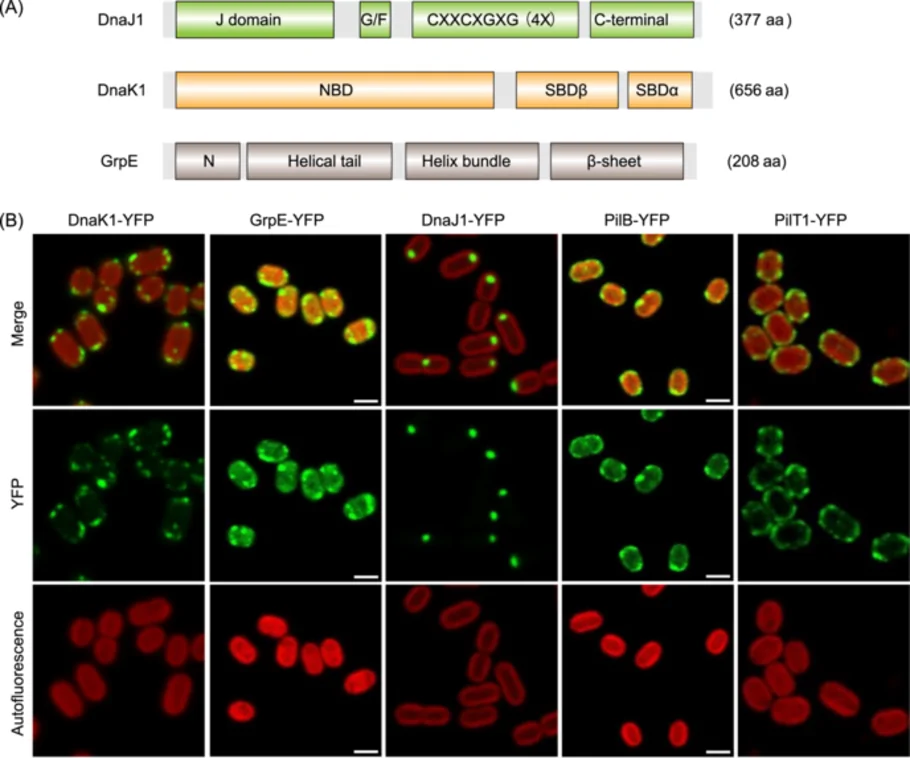
Domain architecture and subcellular localization of the K1J1E chaperone system components in UTEX 3055. (A) Domain organization of DnaJ1, DnaK1, and GrpE. DnaJ1 contains an N‐terminal J domain, a G/F region, a zinc‐finger domain (CXXCXGXG motif), and a C‐terminal substrate‐binding/dimerization domain. DnaK1 comprises an N‐terminal nucleotide‐binding domain (NBD) and a C‐terminal substrate‐binding domain (SBD) with β‐sandwich (SBDβ) and α‐helical (SBDα) subdomains. GrpE features an N‐terminal disordered region, followed by a long α‐helix, a short α‐helix, and a β‐sheet domain. (B) Subcellular localization of YFP‐tagged proteins in the WT background. DnaK1‐YFP, PilB‐YFP, and PilT1‐YFP were expressed under leaky conditions (no IPTG); GrpE‐YFP and DnaJ1‐YFP required induction (400 and 200 µM IPTG, respectively). All constructs were C‐terminally fused to YFP at the chromosomal neutral site (NS1). Images show merged autofluorescence (captured with mCherry filter) and YFP channels (top), YFP signal alone (middle), and mCherry autofluorescence (bottom). Scale bars, 2 μm.

Building on our evidence for physical interactions among DnaK1, PixG_Se,_ and PilB, we hypothesized that these proteins may adopt a common spatial organization within the cell. Many phototaxis proteins assemble into functional clusters at the cell poles^27^, ^32^, ^38^, ^39^, prompting us to examine the localization of the K1J1E chaperone system using functional, chromosomally expressed YFP fusions. Fluorescence microscopy revealed that all three chaperones showed varying degrees of polar enrichment (Figure 4B). DnaK1‐YFP predominantly formed discrete polar foci, closely resembling the PixG_Se_‐YFP localization reported previously^32^. DnaJ1‐YFP typically appeared as a single, bright polar focus that was asymmetrically inherited during cell division (Figure S9), raising the possibility of an active segregation or retention mechanism. GrpE‐YFP displayed a mixed pattern, with a diffuse cytoplasmic signal accompanied by bright, cap‐like polar accumulations (Figure 4B).

Given the physical association observed among PixG_Se_, PilB, and DnaK1, we next asked whether the T4P motor ATPase showed comparable spatial organization. Indeed, both PilB‐YFP and PilT1‐YFP formed membrane‐associated puncta enriched at the cell poles (Figure 4B), closely mirroring the localization patterns of PixG_Se_ and DnaK1. Immunoblot analysis confirmed that each YFP‐tagged fusion protein accumulated primarily as a full‐length product, confirming that the observed localization patterns reflect intact fusions despite the presence of minor degradation bands (Figure S10). The similar polar distribution of K1J1E chaperones, PixG_Se_, and T4P motor proteins is consistent with a model in which these factors assemble at defined cellular sites to coordinate T4P activity and motility, though direct evidence for such assembly remains to be obtained.

## DISCUSSION

Phototaxis allows cyanobacteria to move toward optimal light conditions for photosynthesis, enhancing their survival in dynamic environments. Despite extensive studies exploring light‐induced motility in *Synechocystis* and *Nostoc*, how the signal is transmitted from the phototaxis sensory system to the control of type‐IV pili activity remains largely unknown. In *Synechocystis* PCC 6803, the PATAN‐domain response regulator PixG_Se_ directly interacts with the T4P motor PilB^26^, but whether additional proteins participate in or regulate this complex has remained unclear. Here, we identify the molecular chaperone DnaK1 as a previously unrecognized component at this interface in UTEX 3055, demonstrating that a chaperone physically associates with both PixG_Se_ and PilB. This finding adds a new layer of regulation—the potential involvement of a chaperone in complex stabilization—to the existing PixG–PilB interaction framework. Moreover, while prior work in *N. punctiforme* showed that DnaK1 interacts with PilB and Hfq, and is required for polysaccharide secretion^31^, our study reveals a distinct role for DnaK1 in directly regulating phototactic motility in a unicellular cyanobacterium, although we also observed an associated increase in biofilm formation in the Δ*dnaK1* mutant (Figure S5). Based on these findings, we propose a working model in which a previously unrecognized chaperone system spatially and functionally couples light sensing to directed motility (Figure 5).

**Figure 5:**
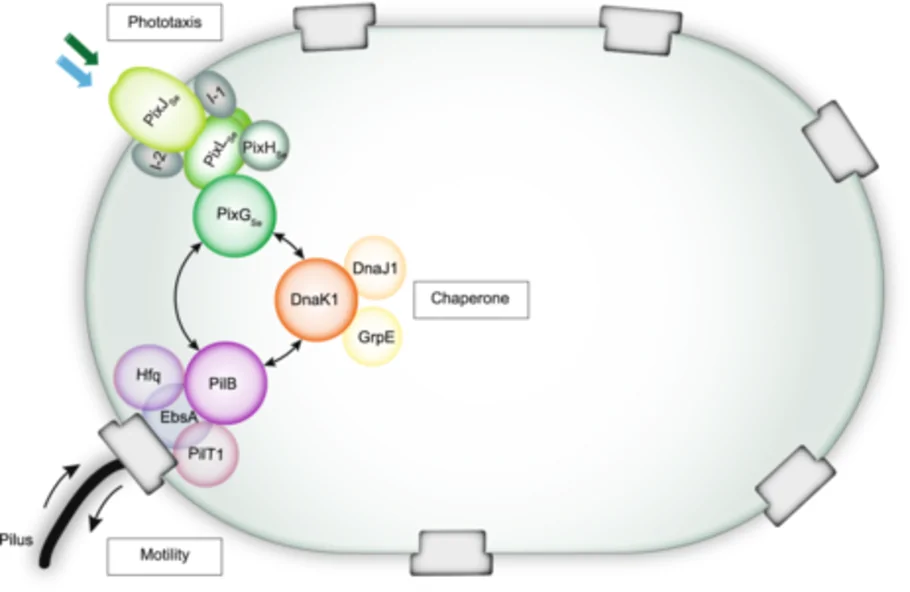
A hypothetical model for DnaK1‐mediated regulation of phototactic motility in UTEX 3055. Environmental light signals are perceived by the photoreceptor PixJ_Se_ and transmitted through the PixL_Se_–PixG_Se_ two‐component system. The response regulator PixG_Se_ functions as a key phototaxis regulator, linking sensory input to type IV pilus (T4P) activity. At the pilus base, motor proteins such as PilB and PilT1, together with proteins including Hfq and EbsA, drive pilus extension–retraction cycles required for motility. Previous studies have shown that PixG directly interacts with PilB in PCC 6803^26^. In UTEX 3055, our biochemical and localization data identify DnaK1, assisted by cochaperones DnaJ1 and GrpE, as a potential regulator at this interface. DnaK1 interacts with both PixG_Se_ and PilB, localizes to the cell poles, and contributes to the regulation of phototaxis and biofilm formation. We hypothesize that DnaK1 may stabilize PixG_Se_ signaling activity and/or facilitates PilB folding and assembly, thereby potentially organizing functional hubs for motility regulation. Through this role, DnaK1 could provide a mechanism for coupling light‐sensing pathways with motor output and may coordinate the motility–biofilm lifestyle switch. I‐1, PixI_Se_‐1; I‐2, PixI_Se_‐2.

The discovery of DnaK1 as a PixG_Se_ interactor expands the functional repertoire of Hsp70 chaperones in cyanobacteria. Unlike the stress‐inducible DnaK2 or the thylakoid‐associated DnaK3^5^, ^40^, DnaK1 is dispensable for growth but important for phototaxis in UTEX 3055. Notably, a role for DnaK1 in motility was previously suggested in unicellular cyanobacteria. While dispensable for cell growth in *Synechocystis*, its deletion severely impairs motility^6^, as well as in *Nostoc*, where DnaK1 regulates HPS production tied to T4P activity^31^. This connection appears to be potentially evolutionarily conserved, as evidenced in *Myxococcus xanthus*, where a nonessential DnaK homolog supports social motility and extracellular matrix production^41^. Together, these observations raise the possibility of an evolutionarily conserved role for DnaK1 in regulating bacterial motility, although the molecular mechanism underlying this regulation remains poorly understood. In this study, our biochemical assays establish that DnaK1 interacts with both PixG_Se_ and PilB. Based on these data, we propose that DnaK1 may stabilize or facilitate the interaction of PixG_Se_ with T4P component PilB. The reciprocal co‐purification of PixG_Se_, DnaK1, and PilB is consistent with, but does not prove, a tripartite complex, suggesting a function of the DnaK1 chaperone system in aiding the formation of a protein complex for phototactic signaling. The requirement of DnaK1 for phototactic motility suggests a context‐specific role in modulating T4P function. PilB's weak interaction with PixG_Se_, as observed in our pulldown assays, may be modulated by phosphorylation state of PixG_Se_, a known regulator of phototaxis signaling^27^, ^32^. One attractive model is that DnaK1 stabilizes phosphorylated PixG_Se_, thereby prolonging its functional lifetime and ensuring efficient recruitment of PilB. Given that phosphorylation often renders regulatory proteins unstable or prone to degradation, DnaK1 could function as a protective chaperone, shielding phosphorylated PixG_Se_ from proteolysis and maintaining its conformational state compatible with PilB engagement. Such a role would parallel the action of Hsp70s in eukaryotic signaling pathways, where chaperone networks stabilize active kinases or transcription factors to sustain signal transmission^42^, ^43^, ^44^. A second, complementary hypothesis is that DnaK1 may be involved in the direct folding, maturation, or conformational priming of PilB itself. Its functional assembly into the T4P motor complex is likely highly demanding in terms of protein quality control. PilB is a large, multi‐domain ATPase that undergoes repeated nucleotide‐driven conformational changes to power T4P‐mediated motility in many bacteria^45^, ^46^. By analogy to previous work showing that DnaK aids the incorporation of cofactors into photosystem components in cyanobacteria^22^, DnaK1 may facilitate the proper folding of PilB's ATPase domains or stabilize PilB during motor assembly at the membrane. This function would ensure that a pool of assembly‐competent PilB is maintained at sites of pilus biogenesis, thereby directly influencing the efficiency of pilus‐driven motility. A third hypothesis concerns spatial organization. The striking polar localization of DnaK1, coincident with both PixG_Se_ and PilB, raises the possibility that DnaK1 is not uniformly distributed in the cytoplasm, but instead targeted to specific subcellular sites where motility regulation occurs. However, we note that co‐localization alone does not establish recruitment. In one working model, DnaK1 could act as a scaffold that concentrates PixG_Se_ and PilB at the poles, promoting the formation of higher‐order regulatory complexes. Direct testing of this model—for example, by examining PixG_Se_ or PilB localization in a Δ*dnaK1* background—will be an important direction for future research. This model is reminiscent of the role of chaperones in bacteria such as *Caulobacter crescentus*, where localized protein complexes orchestrate cell cycle events^47^. By restricting PixG_Se_–PilB interactions to defined cellular zones, DnaK1 may help impose spatial fidelity on the signaling pathway, ensuring that sensory inputs are translated into motility outputs at the correct cellular sites.

Our genetic analysis indicates that DnaK1 and DnaJ1 function together in phototaxis. Based on well‐characterized DnaK1/DnaJ1 systems in other bacteria^48^, ^49^, DnaJ1 likely functions as a cochaperone that stimulates the ATPase activity of DnaK1 and modulates its substrate specificity, though the precise mechanistic basis of their cooperation in this context remains to be determined. In contrast, our data do not allow strong conclusions about the specific role of GrpE. We were unable to obtain a fully segregated Δ*grpE* mutant, as repeated attempts left wild‐type copies persisting even under extended antibiotic selection (Figure 2A). The partially segregated strain displayed only a mild motility impairment. However, because this strain retains wild‐type alleles, this phenotype cannot be unambiguously attributed to a specific role for GrpE in phototaxis. Definitive conclusions await construction of a conditional allele or a fully segregated mutant. Notably, *grpE* encodes the sole nucleotide exchange factor in the chaperone network, whereas multiple *dnaK* and *dnaJ* paralogs exist in *S. elongatus* PCC 7942/UTEX 3055 (Table 1). Our data suggest that *grpE* may be essential, which would be consistent with its unique, nonredundant role as the sole nucleotide exchange factor for all three DnaK paralogs in UTEX 3055, in contrast to the partial redundancy observed among the multiple DnaK and DnaJ paralogs. If confirmed, this essentiality would point to a central role for GrpE in multiple chaperone systems beyond phototaxis. The observed localization of GrpE to both the cytoplasm and cell poles is consistent with potential multifunctionality, as also reported in *Synechocystis*, where GrpE interacts with all three DnaK proteins^10^. It is noteworthy that cyanobacteria generally encode numerous DnaJ‐domain proteins, with at least seven in *Synechocystis* and ten in PCC 7942 (Table 1). We found that DnaJ1 can be deleted without affecting viability under standard conditions. This contrasts with its homolog Sll0897 in *Synechocystis*, which, although dispensable, results in growth defects under normal and especially heat‐stress conditions and has been classified as a canonical HSP^16^. Interestingly, another DnaJ homolog, Sll1384, has been implicated in phototaxis, likely through protein–protein interactions^19^, consistent with the idea that specialized chaperone components may aid T4P‐dependent motility in cyanobacteria.

Beyond cyanobacteria, our findings raise the possibility that chaperone‐assisted regulation of motility may be a feature present in diverse bacteria. In *Pseudomonas aeruginosa*, disruption of *dnaK* reduces flagella‐driven swimming motility^50^, whereas in *Myxococcus xanthus*, a non‐essential *dnaK* homolog supports social motility and extracellular matrix production^41^. These observations are consistent with the idea that the DnaK chaperone system could function as a modulator of certain motility systems, potentially helping to integrate environmental cues with motor output. In UTEX 3055, we additionally uncovered a role for DnaK1 in biofilm formation: The Δ*dnaK1* mutant displayed an approximately fourfold increase in biofilm biomass relative to the WT. This inverse relationship between reduced phototaxis and enhanced surface attachment is consistent with a well‐recognized motile–sessile tradeoff in bacteria^51^. However, we note that our current data do not directly demonstrate that the same DnaK1‐dependent pathway controls both behaviors; the hyper‐biofilm phenotype may represent a separate consequence of DnaK1‐dependent activity rather than a regulated switch. Nonetheless, the observation raises the hypothesis that DnaK1 could influence population‐level lifestyle decisions alongside single‐cell phototaxis. Given that T4P‐dependent motility in cyanobacteria contributes both to directional light responses and to multicellular organization, including biofilm formation and colony spreading^25^, it remains possible that the enhanced biofilm phenotype of the Δ*dnaK1* mutant contributes directly to its phototaxis defect. By linking DnaK1 to both phototactic motility and surface‐associated growth, our results are consistent with a model in which DnaK1 plays a role in coordinating environmental light sensing with the transition between motile and sessile lifestyles, although direct testing of this model will require future work.

Collectively, our findings identify DnaK1 as an important regulator of T4P‐mediated phototaxis in UTEX 3055. Through its interactions with PixG_Se_ and PilB at the cell poles, DnaK1 may act as a signaling scaffold that links sensory input to motility output, although direct evidence for scaffolding function—such as demonstrating DnaK1‐dependent recruitment or spatial organization of PixG_Se_ and PilB—remains to be obtained (Figure 5). The additional observation of increased biofilm formation in the Δ*dnaK1* mutant points to a broader physiological role for this chaperone, but the mechanistic relationship between phototaxis and biofilm remains to be determined. By revealing a role for DnaK1 that extends beyond classical stress responses, our study contributes to the mechanistic understanding of phototaxis regulation and may provide avenues for manipulating cyanobacterial motility for biotechnological applications.

## MATERIALS AND METHODS

### Strains and culture conditions

All *S. elongatus* UTEX 3055 strains used in this study are listed in Table S1. Strains were cultured on BG11 agar plates (0.7% agar) supplemented with 10 mM sodium thiosulfate. Liquid cultures were grown in BG11 medium at 30°C under continuous white light (30 μmol photons·m^−2^ s^−1^) with constant shaking at 120 rpm. Antibiotics were added at the following concentrations whenever needed: kanamycin, 50 µg/ml; spectinomycin, 5 µg/ml; streptomycin, 5 µg/ml; and ampicillin 50 µg/ml.

### Plasmid construction and mutagenesis

Plasmids and primers are listed in Tables S2 and S3. Gene knockout plasmids were constructed by replacing target genes with antibiotic resistance cassettes. For protein expression, plasmids encoding C‐terminal YFP/3× Flag or N‐terminal 3× Flag tags under the control of the P*_trc_* promoter were generated as follows: UTEX 3055 gene fragments were amplified from genomic DNA using primers designed to introduce homologous arms (Table S3). Amplified fragments were ligated into vectors containing a C‐terminal YFP/3× Flag or an N‐terminal 3× Flag tag using Gibson assembly. Constructs were verified by Sanger sequencing and introduced into wild‐type UTEX 3055 via homologous recombination, integrating the expression cassette into a neutral locus.

For natural transformation, UTEX 3055 cultures were grown under continuous white light for at least 3 days to an OD_750_ of 0.5. Exponentially growing cells (4–6 ml) were harvested by centrifugation (6000 rpm, 3 min, room temperature), washed once with fresh BG11 medium, and resuspended in 200 μl of BG11. Approximately 3 µg of knockout or complementation plasmid DNA was added to the cell suspension, which was incubated in the dark at 30°C for 24 h without shaking. Transformants were selected on BG11 agar plates containing 10 mM sodium thiosulfate and appropriate antibiotics. Allelic segregation was confirmed by PCR and Sanger sequencing.

### *In vivo* Co‐IP and MS analysis

Cultures (250 ml) at an OD_750_ of 2.0–3.0 were collected by centrifugation (12,000 rpm, 4°C, 3 min). Cell pellets were resuspended in 2 ml of lysis buffer (50 mM Tris‐HCl, 150 mM NaCl, pH 7.4), supplemented with protease inhibitor cocktail (HY‐K0010; MCE). Cells were lysed using a FastPrep‐24 5 G homogenizer at 6 m/s for 30 s with 0.5 g of Zirconia beads, with two cycles interspersed by a 5‐min incubation on ice. Lysates were centrifuged (12,000 rpm, 4°C, 10 min) to remove beads and debris. The supernatant was divided into 3 equal aliquots, each incubated with 20 µl of pre‐washed Anti‐Flag Magnetic Beads (HY‐K0207‐1; MCE) or GFP‐VHH Agarose (MA108‐25T; ABMagic) for 4 h at 4°C with gentle mixing on a tube rotator. Beads (or agarose) were washed with TBST (50 mM Tris‐HCl, 150 mM NaCl, 0.5% Tween‐20, pH 7.4) until the OD_280_ of the supernatant was <0.05, per the manufacturer's instructions.

For MS analysis, immunoprecipitated samples were washed three times with ice‐cold PBS to remove residual detergent. Bead samples were incubated in reaction buffer (1% SDS; 100 mM Tris‐HCl, pH 8.5; 10 mM TCEP (Tris (2‐carboxyethyl) phosphine hydrochloride); and 40 mM CAA (2‐chloroacetamide)) at 60°C for 1 h for protein denaturation, cysteine reduction, and alkylation. Eluates were diluted 1:1 with water, and proteins were digested overnight at 37°C with 1 μg of trypsin. Peptides were purified using homemade SDB desalting columns, vacuum‐dried, and stored at −20°C until analysis. Liquid chromatography‐tandem mass spectrometry (LC‐MS/MS) was performed on a Q Exactive HF mass spectrometer coupled to an UltiMate 3000 RSLCnano system. MS raw data were processed using MaxQuant (V2.0.1) with the Andromeda database search algorithm. Results were filtered at a 1% false discovery rate (FDR) at both protein and peptide levels. Missing values in the total matrix were imputed, and statistical analysis was performed using a two‐sample *t*‐test (*p* < 0.05) to identify significantly enriched proteins.

### Bacterial adenylate cyclase two‐hybrid assays

The BACTH system was used to investigate protein–protein interactions^52^. Primers and plasmids are listed in Tables S2 and S3. Gene fragments were amplified from UTEX 3055 genomic DNA and cloned into linearized pUT18Ca and pKT25a vectors using Gibson assembly. All the resulting plasmids were verified by Sanger sequencing. The adenylate cyclase‐deficient *E. coli* BTH101 was co‐transformed with pUT18Ca and pKT25a plasmids, and spotted onto LB agar plates containing 150 µg/ml ampicillin, 25 µg/ml kanamycin, 0.5 mM IPTG, and 40 µg/ml 5‐bromo‐4‐chloro‐3‐indolyl‐β‐d‐galactopyranoside (X‐gal). Plates were incubated at 30°C for 48 h to identify interactions via blue colony formation. For the quantitative β‐galactosidase assay, eight colonies from LB plates (containing 150 µg/ml ampicillin and 25 µg/ml kanamycin) were inoculated into 500 µl of LB medium supplemented with 150 µg/ml ampicillin, 25 µg/ml kanamycin, and 0.5 mM IPTG, and incubated at 30°C with shaking for 16 h. ß‐galactosidase activity was measured as described^53^, with activity expressed in Miller units.

### Expression, purification, and *in vitro* pull‐down of recombinant proteins

Plasmids for protein expression are listed in Table S2. pET‐28a (containing a His‐tag or a Strep‐tag encoding sequence) and pAM5431‐3× Flag were used as cloning and expression plasmids. *E. coli* BL21 (DE3) cells harboring recombinant plasmids were grown in LB media at 180 rpm, 37°C for fast and new growth. 50 µg/ml kanamycin, 50 µg/ml ampicillin, or 50 µg/ml streptomycin was added to LB media. Protein expression was induced by the addition of 0.5 mM IPTG at an OD_600_ of 0.4–0.6, and the induced cells wer then further incubated at 16°C, 120 rpm for 18 h.

Cells were harvested by centrifugation and resuspended in 25 ml of lysis buffer (50 mM NaCl, 200 mM Tris‐HCl, pH 8.0, 20% glycerol). Cells were lysed and centrifuged (12,000 rpm, 4°C, 10 min), and the supernatant was incubated with Strep‐Tactin XT resin (IBA‐Lifesciences) or Ni‐NTA resin (Qiagen) on ice for 1 h. Proteins were purified following the manufacturer's protocols. To prepare samples, protein loading buffer (70 mM lithium dodecyl sulfate, 100 mM Tris‐HCl pH 8.5, 10% glycerol, 4 mM EDTA, and 0.025% Coomassie brilliant blue G250) was added to purified proteins and boiled at 99°C for 10 min before loading on 10% sodium dodecyl sulfate polyacrylamide gel electrophoresis (SDS‐PAGE) for western blot analysis.

### Western blotting

Polyclonal antibodies against PixG_Se_, DnaK1, and PilB were generated by immunizing rabbits with purified recombinant proteins (Mabnus Company). Immunoblotting was performed as previously described, with minor modifications^54^. Briefly, UTEX 3055 cells were harvested and resuspended in LDS loading buffer (70 mM lithium dodecyl sulfate, 100 mM Tris‐HCl, pH 8.5, 10% glycerol, 4 mM EDTA, and 0.025% Coomassie brilliant blue G250). Samples were boiled at 99°C for 5 min and centrifuged at 12,000 rpm for 5 min. The resulting supernatant was subjected to SDS‐PAGE, and proteins were transferred to a PVDF membrane using the wet transfer method. Membranes were blocked with 5% (w/v) non‐fat milk in PBS overnight at 4°C. Primary antibodies were diluted 1:1000 in 1% (w/v) non‐fat milk in PBS and incubated with the membranes for 1 h at room temperature. Membranes were then washed three times with PBST (PBS containing 0.05% Tween‐20), 15 min each wash. Secondary antibodies were applied under the same conditions at a dilution of 1:2000. Protein bands were detected using a chemiluminescence imaging system (Tannon 5200), and the images were processed with ImageJ software.

### Phototaxis assay

Phototaxis was assessed on BG11 plates solidified with 0.4% (w/v) agarose and supplemented with 10 mM sodium thiosulfate. Wild‐type and mutant cells grown in liquid BG11 were adjusted to an OD_750_ of 7.0, and then spotted (2‐μl per spot) onto the surface of agarose plates. Plates were incubated in a dark box with a single opening facing a fluorescent lamp (20–150 μmol photons m^−2 ^s^−1^) at 30°C for 5–7 days. For quantitative analysis, light intensities were measured at the plate surface using a light meter and categorized as low (0–35 μmol photons m^−2 ^s^−1^), medium (35–55 μmol photons m^−2 ^s^−1^), and high (55–160 μmol photons m^−2 ^s^−1^). Statistical analyses were performed in GraphPad Prism 10.4.

### Biofilm assay

Freshly grown wild‐type and *dnaK*1 mutant cells were diluted to an OD_750_ of 0.2 in BG11 medium, and aliquots (2 ml) of each strain were transferred to 24‐well plates and incubated statically under standard growth conditions (~30 μmol photons m^−2 ^s^−1^) for 9 days. After incubation, the supernatant was carefully removed, and the wells were gently washed twice with distilled water, followed by PBS. Biofilms were stained with 500 μl of 0.025% (w/v) crystal violet for 5 min at room temperature, and then rinsed thoroughly with PBS to remove unbound dye. To quantify biofilm biomass, the bound crystal violet was solubilized in 1 ml of 95% ethanol, and absorbance was measured at 550 nm. The experiment was performed twice independently, with 12 replicates per strain in each experiment. Data are presented as mean values ± standard deviation (SD) from one representative experiment (*n* = 12).

### Microscopy

For protein localization, UTEX 3055 cultures were grown to an OD_750_ of 0.5 in BG11 medium, and 2‐μl aliquots were immobilized on 1% (w/v) agarose pads for imaging. Fluorescence microscopy was performed using a laser scanning confocal system (Leica TCS SP8), with cyanobacterial autofluorescence captured using mCherry filter settings (excitation: 575/25 and emission: 625/45) and YFP‐tagged proteins visualized using YFP‐optimized filters (excitation: 513/17 nm and emission: 548/22 nm).

## AUTHOR CONTRIBUTIONS

**Shang‐Yu Li**: Conceptualization; data curation; formal analysis; methodology; investigation; validation; visualization; writing—review and editing. **Qing‐Xue Sun**: Investigation; methodology. **Dan‐Qing Huang**: Investigation. **Tao Xie**: Investigation. **Yan Yu**: Investigation. **Cesar A. Valades‐Cruz**: Formal analysis. **Chenliu He**: Investigation; resources. **Xiaoli Zeng**: Conceptualization; methodology. **Yiling Yang**: Conceptualization; formal analysis; funding acquisition; methodology; supervision; writing—original draft; writing—review and editing. **Cheng‐Cai Zhang**: Conceptualization; supervision; writing—review and editing.

## ETHICS STATEMENT

This study did not involve human subjects and animals.

## CONFLICT OF INTERESTS

The authors declare no conflict of interests.

## Supporting information

Supporting file 1.

## Data Availability

All quantitative data supporting the findings of this study, including the source data for the phototaxis analyses and biofilm quantification (e.g., Excel files of absorbance measurements and original microscopy images), are available within the article, its Supporting Information, or from the corresponding author upon reasonable request.
