# Supplementary material for: DnaK1‐mediated regulation of phototactic motility in Synechococcus elongatus UTEX 3055
Source: mLife. 2026 Oct 26;5(5):568–81. doi: 10.1002/mlf2.70109 (PMC13710413; doi:10.1002/mlf2.70109)
Supplement: Supplementary file 1 [file MLF2-5-568-s001.docx]

**Supplementary Information**

**Figure S1**. **Amino acid sequence alignment of DnaK proteins.** Alignment of DnaK homolog RS08885 from UTEX 3055 with DnaK1, DnaK2 and DnaK3 from PCC 7942. RS08885 shows the highest sequence similarity to DnaK1.

**Figure S2. Bacterial two-hybrid analysis of PixG_Se_ interactions**. (A) Interaction of PixG_Se_ with candidate proteins was assessed using the bacterial adenylate cyclase two-hybrid (BACTH) system. Fusions of PixG_Se_ to the T18 or T25 fragments of adenylate cyclase were co-expressed with the indicated candidate genes fused to the complementary fragment. *E. coli* BTH101 cells were spotted on selective medium supplemented with X-gal, and interaction strength was visualized by blue coloration. Positive (T18-Zip + T25-Zip) and negative (T18 + T25) controls are shown. A strong blue coloration was only observed for the PixG_Se_–RS08885 (DnaK1) pair. (B) Interaction of PixG_Se_ with DnaK2 was also tested, but no interaction was detected.

**Figure S3**. **Growth phenotype of K1J1E deletion mutants.** WT, Δ*dnaJ1*, Δ*dnaK1* *and* Δ*grpE* strains were grown in BG11 medium to OD_750_ of 0.5, then were serially diluted 10-fold increments from 2*10^-3^ to 2*10^-7^. Aliquots (2-μl) were spotted on BG11 agar plates and air-dried. Plates were incubated at 30 ℃ under constant illumination of 30 μmol photons/m^2^/s for 16 days before imaging.

**Figure S4**. **Quantitative analysis of phototactic migration under different light intensities.** Relative motility is defined as the migration distance normalized to the wild-type control on the same plate. For convenience, light intensities were categorized as low (0-35 μmol/m²-s), medium (35-55 μmol photons/m²-s), and high (55-160 μmol/m²-s). Relative motility data were analyzed by two-way ANOVA with strain type and light intensity as factors, followed by Šídák's multiple comparisons test. Data are shown as mean ± SD (n = 3-11 biological replicates). Significance thresholds: * p < 0.05, ** p < 0.01, *** p < 0.001, **** p < 0.0001; ns, not significant.

**Figure S5**. **DnaK1 is involved in the regulation of biofilm formation.** (A) Freshly grown WT and Δ*dnaK1* strains were diluted to an OD_750_ of 0.2 in BG11 medium, then 2 mL of culture was transferred to 24-well plates and incubated statically under illumination of 30 μmol photons m^-2^s^-1^ for 9 days. The biofilm attached to the wall was imaged after carefully removing the liquid culture and after the crystal violet staining. Representative images of WT and Δ*dnaK1* from one single experiment with 12 replicates are shown. (B) Quantification of the biofilm from (A). The bound crystal violet was solubilized in 1 mL of 95% ethanol, and absorbance was measured at 550 nm. Significance analysis was performed with unpaired t-test, P<0.0001.

**Figure S6**. **Genomic organization of the *pil-dnaK* gene cluster.** Schematic representation of gene arrangement of the *pilB*, *pilT1*, *pilC*, *grpE*, *dnaK1* and *dnaJ1* gene clusters in *Synechococcus elongatus* PCC 7942 / UTEX 3055, *Nostoc punctiforme* PCC 73102, *Anabaena* sp. PCC 7120 and *Synechocystis* sp. PCC 6803. Homologous genes are indicated by identical colors. Adjacent genes that are not part of this conserved cluster are left uncolored.

**Figure S7. Validation of poly-clonal antibody specificity.** (A) Western blot analysis of protein samples using anti-PixG_Se_, anti-PilB and anti-DnaK1 antibodies. Lanes contain purified recombinant proteins from *E. coli* (PixG_Se_-His, PilB-His, DnaK1-strep), whole-cell lysates from Δ*pixG::3xplag-pixG,* WT::*dnaK1-yfp,* WT::*pilB-yfp,* and WT strain. Expected molecular weights: PixG_Se_ (47 kDa), 3xFlag-PixG_Se_ (50 kDa), PilB (74 kDa), PilB-YFP (102 kDa), DnaK1 (71 kDa), DnaK1-YFP (99 kDa). (B) SDS-PAGE of the samples shown in (A), confirming protein loading.

**Figure S8. Phylogeny of the DnaJ homologs.** Phylogenetic tree comparing the DnaJ homolog (RS08880) from UTEX 3055 with DnaJ homologs from PCC 7942 and *Synechocystis*. Proteins sequences were aligned using MAFFT with default parameters (55). The phylogeny was inferred with IQ-TREE under default settings with 1000 bootstrap replicates, and tree was visualized using interactive Tree of Life (iTOL, https://itol.embl.de/). Purple circles indicate nodes with >50% bootstrap support. See Table 1 for detailed information on each DnaJ homolog.

**Figure S9. Subcellular localization of DnaJ1-YFP.** DnaJ1-YFP expressed from the P*trc* promoter at leaky expression levels forms single fluorescent focus in the wild-type background. Scale bar, 1 µm.

**Figure S10. Immunoblot analysis of YFP-tagged fusion proteins expressed from the neutral site (NSI). Total protein lysates from** Wild-type cells expressing the indicated YFP fusion proteins from the genomic NSI locus were analyzed by immunoblotting using an anti-YFP antibody. Proteins were detected by chemiluminescence. Red arrows indicate the positions of the expected full-length fusion proteins.

**Table S1.** Strains used in this study

| **Strains** | **Description** | **Resistance** | **Source** |
| --- | --- | --- | --- |
| ***E. coli* strains** |  |  |  |
| BL21(DE3) | *E. coli* host strain for recombinant protein production |  |  |
| BTH101 | *E. coli* host strain (cya−) for bacterial adenylate cyclase two-hybrid assay |  | (56) |
| BL21::*pixG* | *BL21* host strain for expressing PixG_Se_-strep protein | Km | This study |
| BL21::*pilB* | *BL21* host strain for expressing PilB-strep protein | Km | This study |
| BL21::*dnaK1* | *BL21* host strain for expressing DnaK1-strep protein | Km | This study |
| BL21::*Flag-dnaK1* | *BL21* host strain for expressing Flag-DnaK1 protein | Sp | This study |
| BL21::*pilB-his-Car* | *BL21* host strain for expressing PilB-his protein with Car resistance | Car | This study |
| BL21::*pixG_dnaK1* | *BL21* host strain for expressing PixG_Se_-strep and 3xFlag-DnaK1 proteins together | Km, Sp | This study |
| BL21::*pixG_pilB* | *BL21* host strain for expressing PixG_Se_-strep and PilB-his proteins together | Km, Car | This study |
| BL2*1*::*pilB_dnaK1* | *BL21* host strain for expressing PilB-strep and 3xFlag-DnaK1 proteins together | Km, Sp | This study |
| BL21::*pixG_dnaK1_pilB* | *BL21* host strain for expressing his-PixG-DnaK1-PilB proteins together | Km | This study |
| **Cyanobacterial strains** |  |  |  |
| *S. elongatus* UTEX 3055 | Wild type |  | UTEX culture collection |
| Δ*pixG* | *pixG* gene knockout mutant | Km | This study |
| Δ*dnaJ1* | *dnaJ1* gene knockout mutant | Km | This study |
| Δ*dnaK1* | *dnaK1* gene knockout mutant | Km | This study |
| Δ*grpE* | *grpE* gene knockout mutant | Km | This study |
| Δ*pilB* | *pilB* gene knockout mutant | Km | This study |
| Δ*pilT1* | *pilT1* gene knockout mutant | Km | This study |
| *wt* ::*dnaJ1-yfp* | *wt* expressing DnaJ1-YFP translational fusion from NS1 | Sp, Sm | This study |
| *wt* ::*dnaK1-yfp* | *wt* expressing DnaK1-YFP translational fusion from NS1 | Sp, Sm | This study |
| *wt* ::*grpE-yfp* | *wt* expressing GrpE-YFP translational fusion from NS1 | Sp, Sm | This study |
| *wt* ::*pilB-yfp* | *wt* expressing PilB-YFP translational fusion from NS1 | Sp, Sm | This study |
| *wt* ::*pilT1-yfp* | *wt* expressing PilT1-YFP translational fusion from NS1 | Sp, Sm | This study |
| Δ*dnaJ1*::*3xFlag-dnaJ1* | *dnaJ1* gene knockout mutant expressing 3xFlag-DnaJ1 from NS1 | Km, Sp, Sm | This study |
| Δ*dnaK1*::*3xFlag-dnaK1* | *dnaK1* gene knockout mutant expressing 3xFlag-DnaK1 from NS1 | Km, Sp, Sm | This study |
| Δ*grpE*::*3xFlag-grpE* | *grpE* gene knockout mutant expressing 3xFlag-GrpE from NS1 | Km, Sp, Sm | This study |
| Δ*pixG*::*3xFlag-pixG* | *pixG* gene knockout mutant expressing 3xFlag-PixG_Se_ from NS1 | Km, Sp, Sm | This study |
| Δ*pixG*::*pixG-3xFlag* | *pixG* gene knockout mutant expressing PixG_Se_-3xFlag from NS1 | Km, Sp, Sm | This study |

**TABLE S2.** List of plasmids used in this study.

| **Plasmid** | **Description** | **Antibiotics** | **Source** |
| --- | --- | --- | --- |
| Δ*pixG* | *pixG* gene knockout plasmid | Km | (23) |
| Δ*dnaJ1* | *dnaJ1* gene knockout plasmid | Km | This study |
| Δ*dnaK1* | *dnaK1* gene knockout plasmid | Km | This study |
| Δ*grpE* | *grpE* gene knockout plasmid | Km | This study |
| Δ*pilB* | *pilB* gene knockout plasmid | Km | This study |
| Δ*pilT1* | *pilT1* gene knockout plasmid | Km | (23) |
| pAM5431(Sp) | Plasmid carrying Ptrc-SpSm-lacI-rrnB and arm sequences for integration at NS1 | Sp | (23) |
| pAM5431_*dnaJ1-yfp* | Derivative of pAM5431-(Sp), carrying Ptrc-*dnaJ1*-*yfp* | Sp | This study |
| pAM5431_*dnaK1-yfp* | Derivative of pAM5431-(Sp), carrying Ptrc-*dnaK1*-*yfp* | Sp | This study |
| pAM5431_*grpE-yfp* | Derivative of pAM5431-(Sp), carrying Ptrc-*grpE*-*yfp* | Sp | This study |
| pAM5431_*pilB-yfp* | Derivative of pAM5431-(Sp), carrying Ptrc-*pilB-yfp* | Sp | This study |
| pAM5431_*pilT1-yfp* | Derivative of pAM5431-(Sp), carrying Ptrc-*pilT1-yfp* | Sp | This study |
| pAM5431_*3xFlag-dnaJ1* | Derivative of pAM5431-(Sp), carrying Ptrc-*3xFlag-dnaJ1* | Sp | This study |
| pAM5431_*3xFlag-dnaK1* | Derivative of pAM5431-(Sp), carrying Ptrc-*3xFlag-dnaK1* | Sp | This study |
| pAM5431_*3xFlag-grpE* | Derivative of pAM5431-strep (Sp), carrying Ptrc-*3xFlag-grpE* | Sp | This study |
| pAM5431_*3xFlag-pixG* | Derivative of pAM5431-strep (Sp), carrying Ptrc-*3xFlag-pixG* | Sp | This study |
| pAM5431_*pixG-3xFlag* | Derivative of pAM5431-strep (Sp), carrying Ptrc-*pixG-3xFlag* | Sp | This study |
| pUT18Ca | BACTH plasmid containing *cyaA* (T18) gene fragment, for insertion of a target gene at C terminal of T18 | Car | (56) |
| pKT25a | BACTH plasmid containing *cyaA* (T25) gene fragment, for insertion of a target gene at C terminal of T25 | Km | (56) |
| pUT18Ca_*zip* | A derivative of pUT18Ca, expressing T18_Zip fusion，positive control for BACTH assay | Car | (56) |
| pKT25a_*zip* | A derivative of pKT25a, expressing T25_Zip fusion，positive control for BACTH assay | Km | (56) |
| pKT25a_*pixG* | pKT25a expressing T25_PixG_Se_ fusion | Km | (24) |
| pUT18Ca_*dnaK1* | pUT18Ca expressing T18_DnaK1 fusion | Car | This study |
| pHStag | pET28a-derived vector for expressing recombinant protein with a C-terminal Strep tag and/or a N-terminal His tag | Km | (57) |
| pET28-*pixG-strep* | pET28a for expressing recombinant protein PixG_Se_, used for protein purification assay with strep tag | Km | This study |
| pET28-*dnaK1-strep* | pET28a for expressing recombinant protein DnaK1, used for protein purification assay with strep tag | Km | This study |
| pET28-*pilB-strep* | pET28a for expressing recombinant protein PilB, used for protein purification assay with strep tag | Km | This study |
| pET28-*car-pilB-his* | Derivative of pET28-PilB-his (Km) for expressing recombinant protein PilB, used for protein purification assay with his tag and Car resistance | Car | This study |
| pET28-*his-pixG-dnaK1-pilB* | Derivative of pET28-PilB-his (Km) for expressing recombinant protein PilB, used for protein purification assay with his tag and Car resistance | Km | This study |

**TABLE S3.** List of primers used in this study.

| **Primers** | **Sequence (5’-3’)** |
| --- | --- |
| PpCVD-F327 | ATACCGCACAGATGCGTAAG |
| PpBR322ori-C3G3-F | GTCCCCGGGCCCGGGCCCGAT |
| PpBR322ori-G5C5-R | GTCCCCCCGGGGGCCCCCGAT |
| PaphI-C2G-F | ATCCGGCGGCGGCGGCGGGAT |
| PaphI-GC-R | ATCGCGCGCGCGCGCGCGGAT |
| Pkan-F | CGCGAGCCCATTTATACCCATA |
| Pkan-R | GCCTCGGTGAGTTTTCTCCTTC |
| Pspsm-F | GTTATGGAGCAGCAACGATG |
| Pspsm-R | TGGGTTCGTGCCTTCATC |
| PRS08880-LA-C3G3-F | atcgggcccgggcccggggacTGCATGTCCTGCAAGGGGAGCGT |
| PRS08880-LA-C2G-R800 | atcccgccgccgccgccggatGGGCTGGATGGGGTCGAGCGA |
| PRS08880-RA-GC-F | atccgcgcgcgcgcgcgcgatGCCCCACCGAGCTGATCATTCCGC |
| PRS08880-RA-C5G5-R800 | atcgggggcccccggggggacTGTAGCGGCTCGAGTTCGGGCTCGG |
| PRS08885-LA-C3G3-F | atcgggcccgggcccggggacATCGCCTGCAATGACTGATTTCATGCTTG |
| PRS08885-LA-C2G-R800 | atcccgccgccgccgccggatAAATTTTTGTGTAGGTGGCTACCTGAAATGATCCT |
| PRS08885-RA-GC-F | atccgcgcgcgcgcgcgcgatGGCGATCGGGGCTGGGG |
| PRS08885-RA-C5G5-R800 | atcgggggcccccggggggacAGTTTCTTCGGCCGCCGTAGAC |
| PRS08890-LA-C3G3-F | atcgggcccgggcccggggacTCCAGATAGCCCGCAATCAGCCGTTC |
| PRS08890-LA-C2G-R800 | atcccgccgccgccgccggatGACGATTGAGAGTCCTACCACAACAAGCTAAACACT |
| PRS08890-RA-GC-F | atccgcgcgcgcgcgcgcgatTCTCTATAGCTCTTAGTAGACTCTTACCCTAGGCTA |
| PRS08890-RA-C5G5-R800 | atcgggggcccccggggggacGTCAACAATGACAGCGTCAAAGTCATCCCCAC |
| PpilB-LA-C3G3-F | atcgggcccgggcccggggacTCCTCGAGCACGATGCCATCCG |
| PpilB-LA-C2G-R800 | atcccgccgccgccgccggatGGGTGGCAGTGGCTGAGAGGAAAAGAT |
| PpilB-RA-GC-F | atccgcgcgcgcgcgcgcgatCAGTCAGCAGCGATCGTGCAAAACCGGCTCGAGTGAGT |
| PpilB-RA-C5G5-R800 | atcgggggcccccggggggacCAATAATCCGGTCAACGGTTTGGGCTGCAGAGCTGGT |
| PpilT1-LA-C3G3-F | atcgggcccgggcccggggacGGCCTTCATGCGGCAGGATCCCGATGTGAT |
| PpilT1-LA-C2G-R800 | atcccgccgccgccgccggatCTTGGGCCTCCTAGGGAGTAACGAGAACTTGAA |
| PpilT1-RA-GC-F | atccgcgcgcgcgcgcgcgatCGTGGTTGCACAGGAGATTGAGTAATGCCGAC |
| PpilT1-RA-C5G5-R800 | atcgggggcccccggggggacAGGTTGTCTACACTGAGGCGGCCAGGTGGAGTTG |
| PpSC208_F5558 | AAATAAATGAAGGAGCTCCCCGGCAGG |
| PpSC208_R5557 | AAATTTTCCTCCTTAGGATCCGCATGCGTG |
| PpAM5431_seqF | cgcgcgaattgatctg |
| PpAM5431_seqR | CCCCGATTCTAGAAAGCTTC |
| NS1LA_F2 | cgatcgcggtcacatcgctatctcttaggacttc |
| NS1RA_R2 | GATGACCTTGAGCAGCTGGGTCTAGTCCTCG |
| RS08880seqF | CAAAGACAACAAGAGTTTGGGACGTTTCCAATTAACTGG |
| RS08880seqR | CAAAAACCAGCAGGATTTGATCGACATT |
| RS08885seqF | CTACGGGAAGAGACGACGGAGCAT |
| RS08885seqR | CCTTGTCTGCGTCGCGAGCAAC |
| RS08890seqF | CCGGTTGAGTTCGTCCAAGGCATCG |
| RS08890seqR | CCTTCGTGGCTTGACGTTGGGCATCGGTA |
| PU3055_pilB_seqUP | CAGATCCACCCATCTCCACCA |
| PU3055_pilB_seqDown | GCTATCTTCAGAGCATAATTCCCAG |
| PU3055_pilT1_seqUP | CGCTGCTTGCGAAGAGC |
| PU3055_pilT1_seqDown | GAACTAACAGTCAGCAGCGAT |
| PpAM5431_RS08880_F1 | tgcggatcctaaggaggaaaatttATGGCTGCTGACTACTACCAACTGCTTGGC |
| PYFP_RS08880_R1128 | caccattccacctcctgaaccTCCGCCAAAGACTCCTTTGAAGAAGCC |
| PpAM5431_RS08885_F1 | tgcggatcctaaggaggaaaatttATGGGCAAGGTTATCGGCATCG |
| PYFP_RS08885_R1965 | caccattccacctcctgaaccCTCAATCGCCTCGTAGTCAGC |
| PpAM5431_RS08890_F1 | tgcggatcctaaggaggaaaatttATGAGCGAACATCAGACCCCTCCAGAGG |
| PYFP_RS08890_R621 | caccattccacctcctgaaccTTCTGTTACCGCTGCAGAATTTTCCTCTGCCG |
| PpAM5431_3xFlag_RS08880F1 | ctggtggtggtagcactagcATGGCTGCTGACTACTACCAACTGCTTGGC |
| PpAM5431-RS08880-R1131 | ttctcctgccggggagctccttcatttatttTCATCCGCCAAAGACTCCTTTGAA |
| PpAM5431_3xFlag_RS08885F1 | ctggtggtggtagcactagcATGGGCAAGGTTATCGGCATCG |
| PpAM5431-RS08885-R1968 | ttctcctgccggggagctccttcatttatttCTACTCAATCGCCTCGTAGTCAGC |
| PpAM5431_3xFlag_RS08890F1 | ctggtggtggtagcactagcATGAGCGAACATCAGACCCCTCCAGAGG |
| PpAM5431-RS08890-R624 | ttctcctgccggggagctccttcatttatttTTATTCTGTTACCGCTGCAGAATTTTCC |
| PpAM5431_3xFlag_pixGF1 | ctggtggtggtagcactagcATGAGTAGATTTTGTGGAATGCAGTTGATGGTG |
| PpAM5431_3xFlag_pixGR1251 | ccactacctccagatccaccCTTGTGCAAATGCTGATTAACGATGTTGAGTAATC |
| PpAM5431_PilB_YFPF1 | tgcggatcctaaggaggaaaatttATGCCAGACCAACCGATCTCTCCC |
| PpAM5431_PilB_YFPR1998 | caccattccacctcctgaaccGTTCCCAAAGCGGGGTGTCAGACAATAG |
| PpAM5431_PilT1_YFPF1 | tgcggatcctaaggaggaaaatttATGGAGCTGATGATTGAAGACCTGATGGAAAAG |
| PpAM5431_PilT1_YFPR1086 | caccattccacctcctgaaccTGCAGCAACCGGAACATTACCGATGAGTC |
| UTEX3055_RS08885F1 | atcacctctagtggtgaaATGGGCAAGGTTATCGGCATCG |
| UTEX3055_RS08885 | gatgtcgatctagatctcCTACTCAATCGCCTCGTAGTCAGC |
| PU3055_0945F1 | atcacctctagtggtgaaATGAGTAGATTTTGTGGAATGCAGTTGATGGTGTACTC |
| PU3055_0945R1254 | gatgtcgatctagatctcCTACTTGTGCAAATGCTGATTAACGATGTTGAGTAATCC |
| PpET28pixG-F1 | gtttaactttaagaaggagatataccATGAGTAGATTTTGTGGAATGCAGTTGATGGTG |
| PpET28pixG-strepR1251 | tgctaccaccaccagaaccCTTGTGCAAATGCTGATTAACGATGTTGAGTAATC |
| PpET28_pilB_His_F1 | aggagatataccatgATGCCAGACCAACCGATCTCTCCC |
| PpET28_pilB_His_R1998 | tggtggtggtgatgatgGTTCCCAAAGCGGGGTGTCAGACAATAG |
| PpET28RS08885STREP-F1 | gtttaactttaagaaggagatataccATGGGCAAGGTTATCGGCATCG |
| PpET28RS08885-STREPR1965 | tgctaccaccaccagaaccCTCAATCGCCTCGTAGTCAGC |
| PpET28pilB-F1 | gtttaactttaagaaggagatataccATGCCAGACCAACCGATCTCTCCC |
| PpET28pilB-strepR1998 | tgctaccaccaccagaaccGTTCCCAAAGCGGGGTGTCAGACAATAG |
| pET28-CarF | aacaccccttgtattactgtttatgtaagcaga |
| pET28-CarR | gaattaattcatgagcggatacatatttgaatgtatttagaaa |
| CarF | atatgtatccgctcatgaattaattcTTACCAATGCTTAATCAGTGAGGCACCTA |
| CarR | aaacagtaatacaaggggtgttATGAGTATTCAACATTTCCGTGTCGCCCT |
| pET-F1 | agatccggctgctaacaaagcccgaaaggaagctg |
| pET-R4493 | atggctgccgcgcggcaccagg |
| pET-PixG-F1 | tggtgccgcgcggcagccatATGAGTAGATTTTGTGGAATGCAGTTGAT |
| pET-PixG-R1254 | ggtatatctccttcttaaTTACTTGTGCAAATGCTGATTAACGATGT |
| pET-DnaK1-F1 | ttaagaaggagatataccATGGGCAAGGTTATCGGCATCGA |
| pET-DnaK1-R1968 | ggtatatctccttcttaaTTACTCAATCGCCTCGTAGTCAGC |
| pET-pilB-F1 | ttaagaaggagatataccATGCCAGACCAACCGATCTCTCC |
| pET-pilB-R2001 | tcgggctttgttagcagccggatctTTAGTTCCCAAAGCGGGGTGTC |
